# Biomarkers of apoptosis

**DOI:** 10.1038/sj.bjc.6604519

**Published:** 2008-08-26

**Authors:** T H Ward, J Cummings, E Dean, A Greystoke, J M Hou, A Backen, M Ranson, C Dive

**Affiliations:** 1 Clinical and Experimental Pharmacology Group, Paterson Institute for Cancer Research, University of Manchester, Manchester, UK; 2 Translational Angiogenesis Group, School of Medical Sciences, University of Manchester, Manchester, UK; 3 Department of Medical Oncology, Christie Hospital NHS Trust, Manchester, UK

**Keywords:** apoptosis, biomarkers, cytokeratins, nucleosomal DNA, multiplex ELISA, circulating tumour cells

## Abstract

Within the era of molecularly targeted anticancer agents, it has become increasingly important to provide proof of mechanism as early on as possible in the drug development cycle, especially in the clinic. Selective activation of apoptosis is often cited as one of the major goals of cancer chemotherapy. Thus, the present minireview focuses on a discussion of the pros and cons of a variety of methodological approaches to detect different components of the apoptotic cascade as potential biomarkers of programmed cell death. The bulk of the discussion centres on serological assays utilising the technique of ELISA, since here there is an obvious advantage of sampling multiple time points. Potential biomarkers of apoptosis including circulating tumour cells, cytokeratins and DNA nucleosomes are discussed at length. However, accepting that a single biomarker may not have the power to predict proof of concept and patient outcome, it is clear that in the future more emphasis will be placed on technologies that can analyse panels of biomarkers in small volumes of samples. To this end the increased throughput afforded by multiplex ELISA technologies is discussed.

A biomarker is a characteristic objectively measured and evaluated to indicate normal or pathogenic biological processes or pharmacologic response. Its potential to enhance translational science progress and accelerate drug development is becoming recognised. Nowhere is this more pertinent than in the complex arena of anticancer drug development, where the rate of compound attrition is high and success rates in the clinic are low (Kola and Landis, 2004). Biomarkers may facilitate rational decision-making during drug discovery and in pre-clinical drug evaluation (Collins and Workman, 2006). In addition, pharmacodynamic biomarkers allow real-time monitoring of drug efficacy and identify early signs of toxicity during clinical drug evaluation, while stratification biomarkers should facilitate selection of patients most likely to respond (Ludwig and Weinstein, 2005).

Suppression of apoptosis is a hallmark of human cancer (Weinstein, 2002) and a desired end point of many targeted therapies is induction of tumour cell death. Mechanism-based therapies under clinical evaluation in oncology may directly induce apoptosis by targeting molecular components of apoptosis regulatory pathways (Taylor *et al*, 2006), or do so indirectly, following drug target modulation that is then coupled to apoptosis. Either way, application of informative, validated biomarkers of apoptosis in clinical trials of anti-cancer therapies is urgently required.

## Apoptosis

Cell death can occur by mechanisms including necrosis, mitotic catastrophe and autophagy (Taatjes *et al*, 2008). However, apoptotic cell death regulators are currently considered to have significant potential as targets for cancer therapeutics. Morphological changes during apoptosis include plasma membrane blebbing, cell shrinkage, chromatin condensation and formation of apoptotic bodies (Makin and Dive, 2001). The biochemistry of apoptosis is summarised in three stages, the activation of initiator caspases, mitochondrial release of ‘apoptogens’ and finally the activation of effector caspases, which cleave recognised substrates to dismantle the dying cell.

Molecularly, apoptosis is activated via either the death-receptor-mediated extrinsic pathway or the mitochondria-directed intrinsic pathway. The extrinsic pathway, triggered by ligands binding plasma membrane death receptors, leads to activation of initiator caspase 8 (Fas *et al*, 2006). In certain cell types, this directly activates effector caspases, such as caspase 3, whereas in others (and in most cancer cells) caspase 8 can amplify death signalling by engaging the intrinsic pathway. The latter is controlled by pro- and anti-apoptotic Bcl-2 family proteins where, upon an apoptotic stimulus, changes in intrafamily protein interactions at the mitochondrial surface determine the release of cytochrome *c*. Cytosolic cytochrome *c* activates the apoptosome complex, initiator caspase 9 and the effector caspases. Caspase cleavage of cytokeratins (CKs), poly(ADP-ribose) polymerase and activation of endonucleases (to generate nucleosomal DNA (nDNA)) form a cascade of irreversible events and lead to the formation of apoptotic bodies. They also promote exposure of phosphatidylserine on the external surface of the plasma membrane, which allows phagocyte recognition of the dying cell.

**Figure 1:**
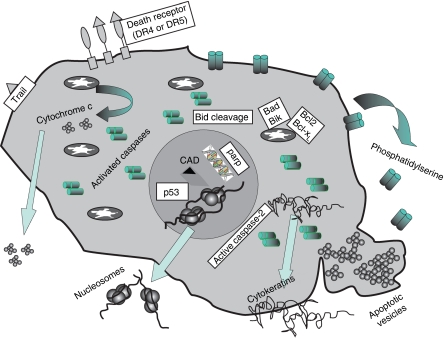
Schematic diagram of the consequential accumulation of proteins following induction of apoptosis. These biomarker molecules are eventually released and can be detected in the circulation in patients undergoing therapy.

Many of the above-mentioned molecular events are potential biomarkers of apoptosis (Figure 1), (Singhal *et al*, 2005) (Table 1). However, detection of apoptosis *in vivo* is challenging; it is generally asynchronous and the half-life of apoptotic cells in tissues is short. Thus, time of analysis is critical regarding the levels of apoptosis detected in patient samples. Apoptosis kinetics are dependent upon the drug's mechanism of action, its pharmacokinetics and critically, the apoptotic threshold of the cells in question.

## Application of biomarkers in clinical trials

Biomarker qualification is the preferred terminology for the evidentiary procedure of causally linking a biomarker to a biological process, pharmacodynamic (PD) effect or clinical end point (Wagner, 2002). This is a lengthy process requiring retrospective and prospective clinical trials and large population screening. This duration may not be necessary for biomarkers intended for early-phase drug discovery (Lee *et al*, 2005). Therefore, qualification and method validation requirements depend upon the inherent assay quantitation and the position of biomarkers in the spectrum toward the clinical end point (Cummings *et al*, 2008). Nonetheless, emphasising on mechanistic studies at the pre-clinical stage with robust PD biomarker assays will increase prospects of successful outcomes in the clinic (Sarker and Workman, 2007).

## Ideal characteristics of a biomarker

Use of PD biomarkers in early clinical trials extends hypothesis testing and confirms whether a drug hits a tumour target (proof of mechanism, POM) and thereafter, the anticipated tumour outcome is reached (proof of concept, POC). The PD biomarkers compared before and after drug treatment may reflect changes in drug target (e.g., protein phosphorylation, direct DNA damage and enzyme activity), or those distal to target hitting (e.g., downstream signalling events, changes in gene expression). Cell fate after target modulation should then be apparent (e.g., changes in proliferation, apoptosis or angiogenesis) (Hidalgo, 2004). Biomarkers measured in tumour and/or surrogate body fluids encompass a broad range of molecules (proteins, nucleic acids, lipids and sugars) as well as circulating intact cells. An ideal biomarker should provide a minimally invasive/non-invasive indirect continuous readout of disease/drug activity.

Ideal biomarkers should therefore aspire to the following criteria:

- Specificity for the biological process/target
- Accurately quantifiable in clinical samples with sufficient dynamic range to detect change upon drug treatment
- Provide a rapid, reliable and robust measurement
- Validateable and validated to internationally recognised standards
- Exhibit little overlap in levels between untreated patients and treated patients
- Have baseline levels not subject to wide variations between patients
- Have levels that correlate with the total burden of disease and unaffected by unrelated conditions
- Have levels that correlate closely with the proximal or distal effects of therapy, thereby aiding POM or POC
- Measurable in a readily obtainable clinical sample

The best approach to chart the progress of apoptosis in a patient's tumour directly before and after a therapeutic intervention would be to interrogate serial tumour biopsies. However, this is usually impractical and unacceptable to the patient. Biomarker analysis procedures that are less invasive must be adopted, including biomedical imaging and detection in more readily obtainable samples such as biological fluids. As the scope of imaging technologies expands and relationships between drug-induced changes in blood-borne biomarkers and tumour images (that detect changes in volume, metabolic activity, and so on) are revealed, a more comprehensive understanding of drug effects in tumours will emerge. There is no ideal biomarker that meets all the above criteria under all circumstances. This is precisely because fulfilling such criteria is so demanding that assays used to measure biomarkers should be validated to exacting standards. Panels of biomarkers must be defined that will in themselves collectively meet these exacting requirements. Such panels may represent a series of single measurements or, more likely, as modern technological platforms emerge, by multiplex measurements. The use of such multiplex measurements will itself generate significant problems for method validation, such as crossreactivity, interference, sensitivity and stability. Such issues will in turn require more complex, although no less stringent, validation strategies. The gulf between technology and the successful deployment of biomarkers in clinical trials is narrowing rapidly, although clinical pharmacology laboratories face significant challenges in implementing biomarker studies in early clinical trials (Cummings *et al*, 2004).

## What we can currently measure

Serial tumour sampling is rarely obtained in early-phase clinical trials, although fixed tissue from diagnosis is often available. As a consequence, the broad utility of tissue biomarkers is limited to prognosis in the first instance and resistance to therapy in the second. Resistance to therapy is often the result of multiple mechanisms such as overexpression of anti-apoptotic proteins, such as Bcl-2 family members (Bcl-2, Bcl-xL, Bcl-w, Mcl-1), or the inhibitors of apoptosis proteins (IAPs), such as survivin or XIAP. In addition, downregulation or mutation of pro-apoptotic proteins, such as Bax, caspase 8, death receptors, p53/p73/p21*waf*I, as well as alterations in NF-*κ*B expression/activity can all contribute to chemoresistance (Igney and Krammer, 2002). Most, if not all, of these proteins have been detected in tissues using immunohistochemistry. However, there are now also many ELISA and cytometric bead-based assays available. The above listed markers remain potential predictors of therapy response rather than truly PD biomarkers, which change in response to therapy. Many of the established caspase substrates are potentially good candidate POC biomarkers of apoptosis that can be measured by immunohistochemistry and ELISA-based assays (e.g., cleaved poly(ADP-ribose) polymerase and cleaved caspase 3). However, if the products of the apoptotic cascade are released into the circulation of cancer patients following therapeutic intervention, they can be more readily measured serially and therefore dynamically. Cytokeratins form approximately 5% of intracellular proteins; therefore, by measuring these, even small numbers of apoptotic cells should be detectable (Biven *et al*, 2003). Specific ELISAs have been developed to quantify CK18 and/or CK19 (e.g., tissue polypeptide antigen, tissue polypeptide-specific antigen and CYFRA21-1) (Holdenrieder *et al*, 2006). These assays are not specific to apoptosis, as necrosis also leads to the release of intact soluble CKs. The M30 apoptosense ELISA uses an antibody to a caspase-cleaved neo-epitope on CK18, whereas the M65 ELISA detects both intact and cleaved soluble CK18. The combined use of M30 and M65 offers potential to dissect mechanisms of cell death in cancer patients (Kramer *et al*, 2004).

Apoptotic endonucleases preferentially cleave DNA between nucleosomes, and the resultant oligonucleosomes are detectable in serum where histones partially protect DNA from further nuclease degradation. In healthy subjects, nDNA has a short half-life; however, their levels are elevated in cancer patients suggesting high levels of production, altered catabolism or both (Holdenrieder *et al*, 2006), and ELISA assays can detect nucleosomes in cancer patient sera. Since CKs do not provide any information on cell death from non-epithelial cells, their combined use with nDNA provides a biomarker panel to assess caspase-dependent and -independent cell deaths of all nucleated cells.

Such assays are being integrated into trials of pro-apoptotic therapies as POC biomarkers (Cummings *et al*, 2006). Although these biomarkers are elevated in cancer patients, they are not sufficiently specific for diagnosis. However, high levels appear to be associated with poor prognosis in certain tumour types, probably reflecting tumour burden. Indeed tissue polypeptide antigen and tissue polypeptide-specific antigen have been used as tumour markers (Holdenrieder *et al*, 2006; Ulukaya *et al*, 2007), and increases in their levels after chemotherapy may be associated with therapeutic response, although this has not been consistently reproduced (Kramer *et al*, 2004; Olofsson *et al*, 2007; Ulukaya *et al*, 2007). In one of the largest studies, nDNA and CYFRA21-1 were measured in 311 patients with NSCLC receiving chemotherapy (Holdenrieder *et al*, 2006). Changes in nDNA and CYFRA21-1 predicted response independently from stage and performance status. These assays in combination demonstrated 100% specificity for response with a sensitivity of 29%, suggesting that they add clinically meaningful information to patient management. Clearly, panels of multiple validated biomarkers specifically tailored to particular treatment regimes or disease groups are the way forward. Currently, we are using existing tumour markers to follow dynamic fold changes in biomarker levels in response to therapy, a hitherto little explored approach. However, new biomarkers as predictors of response as well as patient survival are urgently needed. Such novel biomarkers will need to be tested in large trials with full clinical data available and follow-up, and the biomarker data subjected to rigorous statistical evaluation before implementation into routine clinical practice. The goal of stratifying patients based on biomarker expression is yet to be fulfilled and is an exciting goal for moving ahead.

## Biomarkers: the clinical challenges

Cancer is complex and it is increasingly recognised that tumour cells are rarely addicted to a single pathway, and therefore targeting a single pathway is unlikely to be effective in producing durable remissions due to plasticity, redundancy and feedback mechanisms within molecular signalling pathways. Likewise, considering ‘biomarkers’ as a generic term is an oversimplification. Biomarkers that identify at-risk individuals, detect disease earlier, determine prognosis, detect recurrence/metastases and predict or monitor response/toxicity to treatment are needed (Voorzanger-Rousselot and Garnero, 2007). It is essential that targeted therapies and their associated biomarkers co-evolve.

One key barrier is the lack of high-quality reference material to define biomarker normality, as before you can detect an abnormality, it is essential to know the normal range of a biomarker. Moreover, to get an understanding of the biomarker dynamic range in tumours (given tumour heterogeneity) requires comprehensive international databases of healthy individual and cancer patient samples, collected by standardised methods at multiple time points, analysed retrospectively and prospectively, using validated protocols with quality controls combined with long-term clinical data. Currently, due to interinstitutional variability, this is barely achievable within a nation. Only with this international co-operation, may a consensus of ‘normal’ be obtained and biomarkers discovered, validated to identify false-positives and -negatives, and qualified rapidly.

In current practice, it is extremely rare that biomarker changes accurately represent all of the effects of a therapy on the clinical outcome and, thus, it is essential that biomarker qualification does not distract from robust clinical end points. Finally, as stated above, although many biomarkers correlate statistically with disease end points, this does not automatically prove clinical usefulness (Kattan, 2003). Before integration into the busy clinic, ‘novel’ biomarkers must demonstrate added value beyond that which is already available.

## New technology platforms: the promise of the future

Protein microarray technology is a rapidly evolving field, driven by the need for high-throughput methodology to measure multiple biomarkers in clinical samples. Nearly all the fully tested and characterised protein microarrays are based on antibody technologies. These multiplex platforms have become widely used in the exploratory research arena. To date, three main formats exist: substrate-anchored sandwich ELISA, liquid-based bead assays and protein arrays. Although much effort has been invested in optimisation and instrumentation, these techniques are still only at an early stage of development for use in clinical trials.

ELISAs use an immobilised antibody to capture a soluble ligand, with subsequent detection of captured ligand by a second antibody linked to a reporter molecule. Multiplex plate-based sandwich ELISAs, such as the MSD Mesoscale® (Gaithersburgh, MD, USA) and SearchLight® (Fishers Scientific, Pittsburgh, PA, USA) chemiluminescent arrays are capable of quantification of up to 16 different proteins in multi-well plates. This approach has the advantage that a greater number of analytes can be measured in the same or smaller volume of blood than a conventional single-plex ELISA. This economic use of samples represents a significant saving in both cost and blood/tumour lysate volumes (Nielsen and Geierstanger, 2004). In bead-based assays, such as Luminex and Bead array, capture antibodies are conjugated to polystyrene beads that are uniquely tagged with a combination of two fluorescent dyes. Such dye combinations represent a unique signature for each bead. A second detection antibody tagged with a common fluorochrome is used to quantify the amount of analyte bound to each bead. Detection and quantification are achieved using conventional flow cytometry or dedicated bead-based bioanalysers. Multiplex assays can be created by mixing bead sets with different conjugated capture antibodies to simultaneously test for many (up to 50 or more) analytes in a single clinical sample (Elshal and McCoy, 2006). There are several substantial differences between multiplex platforms in current use and little published work exists regarding validating the relative performances of each platform. Most of the comparisons that have been published to date compare the ‘gold standard’ single-plex ELISA assay to that of a new multiplex system. Comparisons between multiplex and single-plex platforms tend to show good correlations (0.6–0.96). Moreover, both intra- and interassay variations are generally less than 16% (Elshal and McCoy, 2006). The main stumbling block with such comparisons is the source of the antibodies used to capture the analyte, the nature of the capture surface, crossreactivity of the antibodies and heterophilic reactions within the physiological matrix. If comparisons are made between platforms that use identical antibody pairs and detection reagents, correlations tend to be tight. However, even in this scenario, the nature of the substrate is important. Bead-based assays tend to have a lower available surface area to react with analytes than do microspot-based assays. As they flow through the analysis system, beads often have only a half or less of their surface area presented to the signal detector at any time. The uniform, high-density signal from microspots leads to lower levels of detection but higher levels of sensitivity, when compared with bead-based platforms. Despite these issues, multiplex assays are still capable of detection in the analyte nanogram range and a number of multiplex assay platforms have been approved by the FDA (Ling *et al*, 2007).

Protein arrays consist of a large number of regularly arranged discrete microspots of capture molecules, which are transferred onto a solid support using spotting robots. Spotted capture molecules may also be conjugated to fluorescent beads, thereby enabling existing cytometric bead-based technologies to capture nucleic acids, proteins and soluble receptors/ligands. Purified recombinant proteins, antibodies, antibody fragments, aptamers, peptides, nucleic acids or complex protein extracts have all been used as capture molecules. These arrays measure relative protein abundance and are analogous to the DNA arrays commonly used in expression profiling. Comparison of different biological samples in this way is increasingly important in the discovery of potential biomarkers and new targets for therapies.

The development of stable, intensely fluorescent reporter molecules, such as quantum dots, will enhance the multiplexing capacities of protein microarrays (Kersten *et al*, 2005).

Currently, analysis of a single analyte with a single assay is the predominant method applied to most clinical trials. However, once validation strategies have proved multiplex assays to be as robust, reliable and reproducible as single-plex assays, they are destined to comprise a significant part of clinical trial activities. Potentially, these technologies could rapidly trawl through a subset of trial samples to identify the most informative biomarkers to be implemented in the context of that trial. However, one must always bear in mind that regardless of the method chosen to measure biomarkers (singly or multiply), they still exist in a complex biological matrix where they have the potential to interact. More importantly, the rate at which individual biomarkers degrade within such a matrix may vary significantly between biomarkers. Such possibilities mean that analysis of clinical samples should take place as soon as possible following collection. When the path for regulatory compliance for these assays is defined, the development of multiplex systems should accelerate and these approaches should be widely taken up for clinical trials.

## Serial collection of circulating tumour cells: a cell-based apoptotic biomarker?

Circulating tumour cells (CTCs) have been detected in the peripheral blood of patients with solid carcinomas (Cristofanilli *et al*, 2004). Although the development of apoptotic biomarkers has been predominantly focused on the molecular level, decreased CTC numbers may represent an apoptosis-associated biomarker. With the advent of automated, standardised technologies, further morphological and molecular characterisation of CTCs can be carried out in great detail. Accumulating evidence shows that CTCs represent a heterogeneous cell population, among which there exist both apoptotic cells and viable cells with metastatic potential. At the cellular level, the change of CTC number pre- and post-treatment correlates well with patient response to treatment in several cancer types (Cristofanilli *et al*, 2004). Persistence of CTCs 3–4 weeks following therapy strongly suggested that the patients are relapsing with drug-resistant disease and additional chemotherapy would be futile (Cristofanilli *et al*, 2004). At the level of morphology, incorporating Wright–Giemsa staining into the protocol allows fibre-optic array scanning technology to be applied. This approach has shown that CTCs detected from widely metastatic breast cancer patients exhibited early and late apoptotic changes (Marrinucci *et al*, 2007). Further studies have shown that circulating breast cancer cells are frequently apoptotic based on CK staining pattern, nuclear condensation and DNA strand-breaks (Mehes *et al*, 2001), and caspase-cleaved CK18 was detected in circulating prostate tumour cells (Larson *et al*, 2004). In contrast to CT scans, which are expensive, and biopsies, which are difficult to serially collect, the assessment of CTCs provides a readily accessed and cheaper biomarker. Such biomarkers inform on early dynamic changes in the tumour population, which in turn help to evaluate therapeutic response and provide POM for novel pro-apoptotic drugs. Isolation of viable, intact CTCs in sufficient purity and quality may allow informative genomic profiling. Although CTCs may provide a practical useful source of biomarkers, it is not yet known precisely how they relate to the primary tumour, or which CTC markers might predict which cells will metastasise. Technologies for isolating CTCs are advancing rapidly and CTCs have a great potential for biomarker research.

## Summary

In the era of molecular targeted agents, cell death pathways have become key players in drug discovery portfolios. Proteomics can be seen to be playing an ever-increasing role in both the discovery and measurement of biomarkers pertinent to cell death pathways. Apoptosis, therefore, represents not only a vital target in oncology but also a unique biomarker opportunity hitherto unexploited. The challenge ahead lies in discovering new biomarkers and understanding the biology of cellular release of existing apoptotic biomarkers into the circulation. More complex still is the relationship between clinical efficacy, biomarker measurement and overall survival of patients treated with novel molecular targeted agents. As with genomics, key technology tools are now available to fully exploit these opportunities and take apoptosis biomarkers to the forefront of drug discovery and future clinical trials.

## Figures and Tables

**Table 1:** Most commonly described biomarkers of apoptosis can be measured in tissues and blood using a variety of technology platforms

**Biomarker**	**Matrix**	**Analysis platform**	**Comment**
Activated caspases 2, 3, 7, 8 and 9	Tissue	IHC, Elisa, flow cytometry, cytometric bead arrays	Detection by immunoreaction or substrate/active site interactions
Cytochrome *c*	Tissue, serum	ELISA	Useful biomarker measured serially in blood samples
Externalised phosphatidylserine	Cells	ELISA, flow cytometry	Measures Annexin binding to externalised ligand. Early apoptosis event
Cytokeratins	Tissue, serum plasma	ELISA, IHC, Flow cytometry	Useful biomarker measured serially in blood samples
Nucleosomal DNA	Tissue, serum	ELISA, DNA array, PCR	Nucleosomal DNA can be measured serially in serum samples. Extracted DNA can be analysed using PCR
Apo-1/Fas, Fas ligand (sFasL)	Serum, follicular fluid, cells	ELISA, flow cytometry, IHC	Expressed on B and T cells as well as in normal and tumour tissue
Bcl-2/Bcl-xl/Mcl-1	Cells, tissue	IHC, ELISA, flow cytometry	Overexpression contributes to chemo-resistance.
p53, phospo-p53, p21^wafi^, pH2AX	Cells, tissues	IHC, flow cytometry, ELISA	Activation and stabilisation of these proteins informs on DNA damage and repair

IHC=immunohistochemistry; PCR=polymerase chain reaction.

Currently, ELISA platforms and flow cytometry offer the highest throughput for clinical trial use.
